# Solvent Effect on Cation⊗3π Interactions: A First-Principles Study

**DOI:** 10.3390/molecules29215099

**Published:** 2024-10-29

**Authors:** Liuhua Mu, Jie Jiang, Xiao-Yan Li, Shiqi Sheng

**Affiliations:** 1 School of Physical Science and Technology, Ningbo University, Ningbo 315211, China; muliuhua@ucas.ac.cn (L.M.); jiangjie1@nbu.edu.cn (J.J.); 2 Wenzhou Institute, University of Chinese Academy of Sciences, Wenzhou 325001, China; 3 School of Physical Science, University of Chinese Academy of Sciences, Beijing 100049, China; 4 Department of Chemistry, Northwestern University, Evanston, IL 60208, USA; xiaoyan.li@northwestern.edu; 5 School of Physics, East China University of Science and Technology, Shanghai 200237, China

**Keywords:** cation⊗3π interaction, solvent effect, aromatic box, first-principles calculation

## Abstract

Cation⊗3π interactions play a special role in the behaviors of biological molecules and carbon-based materials in aqueous solutions, yet the effects of solvation on these interactions remain poorly understood. This study examines the sequential attachment of water molecules to cation⊗3π systems (cation = Li⁺, Na⁺, K⁺), revealing that solvation influences interaction strengths in opposing ways: solvation of the metal cation decreases the strengths of cation⊗3π interactions, while the solvation of the benzene molecule increases the strengths of cation⊗3π interactions, compared with the strengths of cation⊗3π interactions in the gas phase. The mechanism analyses revealed that in the presence of surrounding water molecules, the stability of cation⊗3π systems is generally enhanced by cation–π, π–π, water–π, and water–ion interactions, while water–water interactions typically have a destabilizing effect. In addition, the primary effect of water molecules at different adsorption sites is to modulate the Coulombic multipole–multipole interactions and the overlap between monomeric charge distributions, thereby influencing the changes in strengths of cation⊗3π interactions. Moreover, AIMD simulations further underscore the practical significance of cation⊗3π interactions. These findings provide valuable insights into the structures and the strengths of cation⊗3π interactions with the effect of solvation.

## 1. Introduction

Cation⊗3π, a term introduced in theoretical studies [1], is hypothesized to play a special role in biological systems and carbon-based materials, where one cation simultaneously binds with three separate aromatic rings. It is different from cation–π interactions due to the cooperative effect between cation–π and π–π interactions, with its stability arising from the synergistic balance among these various interaction types. The conformation of aromatic rings in cation⊗3π interactions is a stable triangular structure, ◬, similar to the aromatic box, which was considered a common structural motif for cation–π interactions [2] and a recurring motif in proteins for the recognition of cationic moieties [3]. The stability of the configuration of aromatic rings in cation⊗3π interactions can be represented by the one-benzene binding (OBB) energy [1]. This calculation assumes that the benzene molecules are identical (e.g., in an equilateral triangular configuration); any irregularity in the triangular configuration may introduce errors. However, the aromatic box is composed of three or more aromatic subunits arranged in a box-like configuration. In addition to the equilateral triangular configuration, the aromatic box can also adopt irregular shapes. Therefore, while the study of cation⊗3π interactions should effectively contribute to our comprehension of the stability of aromatic boxes with regular geometries, it may introduce errors when applied to understanding the stability of aromatic boxes with irregular shapes.

Moreover, we note that the aromatic box can effectively encapsulate positively charged cations and achieve strong cation–π interactions through the cooperativity effects of multiple aromatic side chains [4], and these multiple aromatic rings typically involve π–π interactions [5,6]. Recent theoretical and computational studies [7] have highlighted the importance of cooperativity effects in systems where cation–π and π–π interactions coexist. These effects can significantly alter the interaction energies and overall stability of the aggregates. For instance, it has been observed that the presence of cations can enhance the π–π interaction energy, while the cation–π interaction energy is also slightly increased in the presence of π–π interactions [8,9]. Clearly, the cooperative effect of the interaction between the cation with the three aromatic rings, along with the π–π interactions among the three aromatic rings, could result in strong cation⊗3π interactions. Such strong interactions, obviously, are expected to have a profound influence on the biological function of the biological systems. For example, in 2007, Hughes et al. [10] reported that the recognition of methylated lysine by an aromatic box is mediated by cation–π interactions between the methylated ammonium group and the aromatic box composed of three aromatic residues. In 2009, Xiu et al. [11] demonstrated that nicotine has a high affinity for the acetylcholine (ACh) receptors in the brain because of the strong cation–π interactions between nicotine and the aromatic box of the ACh binding site. Cation⊗3π interactions should also play a significant role in these biological systems due to the existence of steric exclusion effect between the aromatic rings in the aromatic box.

The aqueous solution is an important and abundant component in biological systems, which is a major factor modulating the strength of cation–π interaction in the presence of surrounding solvent [7,12,13,14]. In 1993, Kumpf and Dougherty revealed that the trends of the cation–π interactions in the gas phase are quite different from those in the solvent phase [15]. In 1999, Cabarcos et al. studied the competition between cation–benzene and hydrogen bond interactions and revealed that benzene could effectively replace water molecules to directly interact with K^+^, whereas the primary solvation shell of Na^+^ could not be easily replaced by benzene [16]. Further, in 2009, a systematic computational study of solvent effect on the cation–π interactions between metal cations and a benzene molecule discovered that solvation of the metal cation can weaken the cation–π interactions, while solvation of the aromatic ring can enhance the cation–π interactions [17]. It suggests that the solvent may also enhance the cooperative cation⊗3π interactions and the structural stability of the aromatic box with a cation positioned at its center. Although there are more observations [18,19,20] focused on a cation surrounded by three or more π–electron-rich structures (i.e., the aromatic box), the conformations only contained one or two water molecules bound directly to the cations, and the analyses were still based on the single cation–π interaction. The effect of solvation on cation⊗3π interactions is still far from being fully understood.

In this article, we performed systematical first-principles calculations to explore the effect of solvent (H_2_O) on the cation⊗3π interactions via the selective hydration of the cations or benzene molecules. The results show that the presence of solvent molecules around the metal cation diminishes the strength of cation⊗3π interactions, while solvation of the benzene molecule enhances these interactions relative to their gas-phase values. The mechanism analyses revealed that in the presence of surrounding water molecules, the stability of cation⊗3π systems is generally enhanced by cation–π, π–π, water–π, and water–ion interactions, while water–water interactions typically have a destabilizing effect. The quantum mechanical investigations are expected to provide valuable insights into the structures, and the strengths of cation⊗3π interactions as water molecules are sequentially added along different orientations.

## 2. Results and Discussion

### 2.1. Energetic Analysis

**Figure 1:**
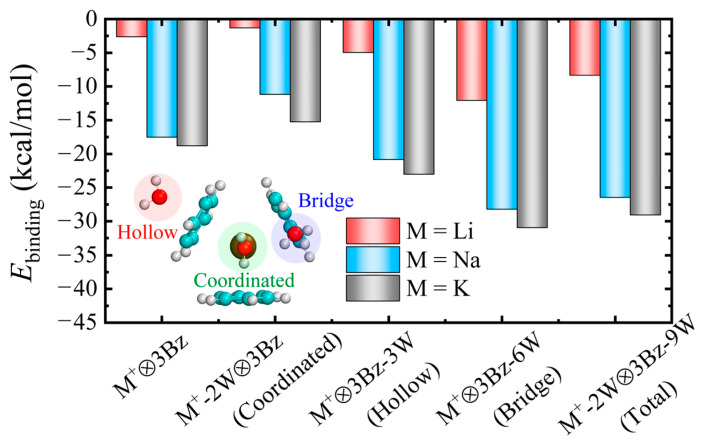
Theoretical computations. OBB energies of (M^+^-*m*W)⊗3Bz-3*n*W (*m* = 0 or 2, *n* = 0, 1, 2, or 3) complexes for M = Li, Na, K. Inset: Schematic depiction of the three hydration sites for the water molecules in the hydration shell of cation or benzene molecules. These hydration sites are indicated by the hollow, bridge, and coordinated sites, respectively.

In order to evaluate the effect of solvation on the cation⊗3π interactions, we performed first-principles calculations via the selective hydration of the cations or benzene molecules in the cation⊗3π system. Firstly, one putative hydration site of cation (denoted as coordinated) and two putative hydration sites of benzene molecules (denoted as hollow and bridge) are considered for introducing the water molecules in the vicinity of the cation⊗3π system, as shown in the inset of Figure 1. Then, all these three hydration sites are also considered (denoted as Total). For the convenience of description, we denote (M^+^-*m*W)⊗3Bz-3*n*W as the cation and each benzene hydrated with *m* and *n* water molecules in the cation⊗3π system, respectively. Here, *m* = 0 or 2, and *n* = 0, 1, 2, or 3. Cation, benzene molecules, and water molecules are denoted as M^+^, Bz, and W, respectively.

**Figure 2:**
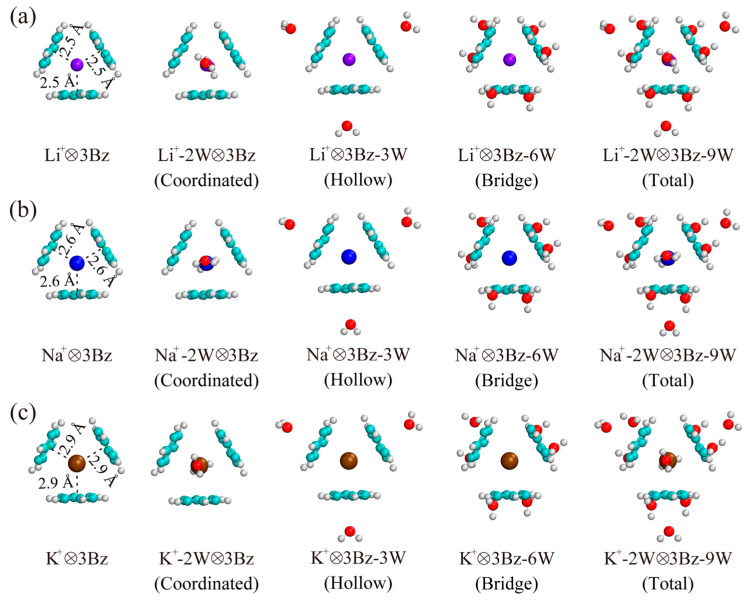
Optimized structures of (**a**) (Li^+^-*m*W)⊗3Bz-3*n*W, (**b**) (Na^+^-*m*W)⊗3Bz-3*n*W, and (**c**) (K^+^-*m*W)⊗3Bz-3*n*W complexes obtained at the MP2/6-311++G(d, p) level. Spheres in purple, blue, brown, cyan, white, and red represent Li^+^, Na^+^, K^+^, carbon, hydrogen, and oxygen, respectively.

**Figure 3:**
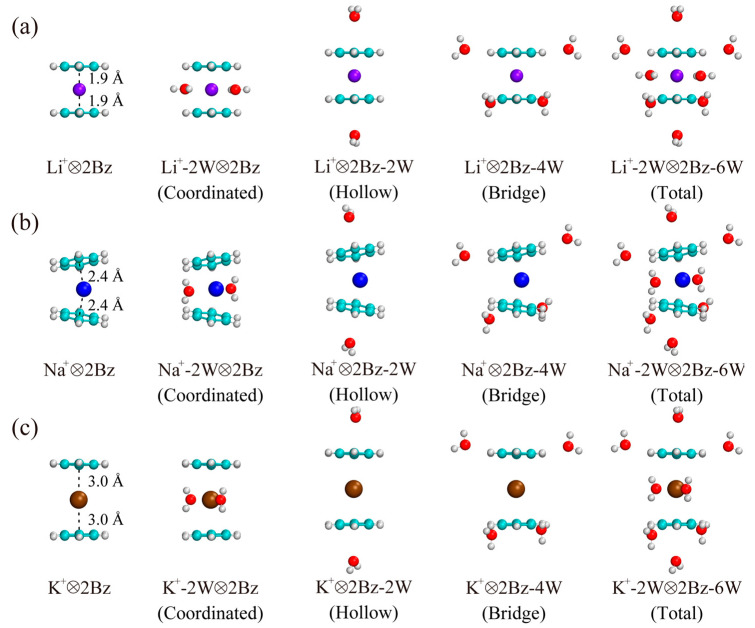
Optimized structures of (**a**) (Li^+^-*m*W)⊗2Bz-2*n*W, (**b**) (Na^+^-*m*W)⊗2Bz-2*n*W, and (**c**) (K^+^-*m*W)⊗2Bz-2*n*W complexes obtained at the MP2/6-311++G(d, p) level. Spheres in purple, blue, brown, cyan, white, and red represent Li^+^, Na^+^, K^+^, carbon, hydrogen, and oxygen, respectively.

The structures of (M^+^-*m*W)⊗3Bz-3*n*W complexes are optimized at the MP2/6-311++G(d, p) level of theory, as shown in Figure 2 (the optimized structures for (M^+^-*m*W)⊗2Bz-2*n*W complexes are shown in Figure 3). To study the stability of the (M^+^-*m*W)⊗3Bz-3*n*W complexes, we calculated OBB energy where each benzene molecule hydrated with *n* water molecules, defined by

(1) $$\begin{array}{c} E_{\mathrm{binding}}\left(\left(M^{+}-mW\right)⊗3\mathrm{Bz}-3nW\right)=E\left(\left(M^{+}-mW\right)⊗3\mathrm{Bz}-3nW\right)\\ -E\left(\left(M^{+}-mW\right)⊗2\mathrm{Bz}-2nW\right)-E\left(\mathrm{Bz}\right)-n\times E\left(W\right) \end{array}$$

where *E*((M^+^-*m*W)⊗3Bz-3*n*W), *E*((M^+^-*m*W)⊗2Bz-2*n*W), *E*(Bz), and *E*(W) are the total energies of optimized (M^+^-*m*W)⊗3Bz-3*n*W complexes, (M^+^-*m*W)⊗2Bz-2*n*W complexes, a single benzene, and a single water molecule, respectively. *E*_binding_((M^+^-*m*W)⊗3Bz-3*n*W) denotes the binding energy between single benzene, *n* water molecules and the residual stable (M^+^-*m*W)⊗2Bz-2*n*W complexes, which can describe the energy required to separate the benzene and *n* water molecules from the stable (M^+^-*m*W)⊗2Bz-2*n*W complexes and result in the (M^+^-*m*W)⊗2Bz-2*n*W complexes together with free benzene and *n* free water molecules.

Figure 1 and Table 1 show the OBB energies with respect to the hydration sites. As expected, in the gas phase, the OBB strengths (the absolute value of the OBB energy) of the M^+^⊗3Bz complexes follow an increasing order, Li^+^ < Na^+^ < K^+^, consistent with the previous studies [1]. For (M^+^-2W)⊗3Bz complexes with two water molecules bound directly to the cations (coordinated site), the OBB strengths increase in the order of Li^+^ < Na^+^ < K^+^, and are significantly weaker than that in the gas phase for M = Li, Na, and K. We note that the larger the value of the interaction energy, the weaker its strength. Interestingly, as water molecules hydrated benzene molecules, the OBB strengths still increase in the order of Li^+^ < Na^+^ < K^+^ for both hollow and bridge sites and are significantly stronger than that in the gas phase. These results indicate that, depending on the site of solvation, the strength of cation⊗3π interaction (OBB strength) becomes stronger or weaker. The enhancement of OBB strengths is similar to the previous observation that the solvation of the aromatic ring enhances the cation–π interactions between cations and benzene molecules [17]. We note that water molecules are important and abundant components in biological systems, and the enhancement of the cation⊗3π interactions in the effect of solvation implies that hydrated cation⊗3π complexes are quite stable and important in biological systems. Moreover, OBB energies for Li^+^⊗3Bz (−2.63 kcal/mol), Na^+^⊗3Bz (−17.53 kcal/mol), and K^+^⊗3Bz (−18.78 kcal/mol) in the gas phase are notably smaller (i.e., more negative) than the previous results (1.31 kcal/mol, −12.08 kcal/mol, and −14.60 kcal/mol for Li^+^⊗3Bz, Na^+^⊗3Bz, and K^+^⊗3Bz obtained using GAUSSIAN-09 software, respectively), as shown in Table 1. This discrepancy may be attributed to differences in quantum chemistry software and structural optimization methods.

It is important to note that the OBB strength of Li^+^ is significantly weaker than that of Na^+^ and K^+^ in the M^+^⊗3Bz complexes (Table 1). As shown in the structures from Figure 2 and Figure 3, the steric exclusion effect between the two benzene molecules in Li^+^⊗2Bz is relatively weak, with the distance between the cation and the centroid of the benzene rings being 1.9 Å. However, in the case of Li^+^⊗3Bz, this distance increases noticeably to 2.5 Å, indicating a strong steric exclusion effect between the three benzene molecules, which pushes them further apart. This pronounced steric exclusion in Li^+^⊗3Bz significantly weakens the OBB strength. In contrast, the distance between the cation and the centroid of benzene in Na^+^⊗2Bz increases only slightly from 2.4 Å to 2.6 Å in Na^+^⊗3Bz. For K^+^⊗2Bz, the distance actually decreases slightly from 3.0 Å to 2.9 Å in K^+^⊗3Bz. These results indicate that the steric exclusion effect between the three benzene molecules is relatively small in Na^+^⊗3Bz and K^+^⊗3Bz, which is consistent with the observed stronger OBB strengths in these systems. Therefore, the steric exclusion effect arises from the spatial arrangement of the ligands around the cation and is crucial in determining the stability and interaction strength within these complexes. In the case of Li^+^, the strong steric repulsion when three benzene molecules are present can lead to unfavorable spatial configurations, thus diminishing the binding strength. Conversely, the relatively small changes in the distance for Na^+^ and K^+^ indicate that their larger ionic radii allow for more favorable steric arrangements, resulting in enhanced OBB strengths.

### 2.2. Mechanism Analysis

In order to elucidate the mechanism underlying the difference in the behaviors between (Li^+^-*m*W)⊗3Bz-3*n*W, (Na^+^-*m*W)⊗3Bz-3*n*W, and (K^+^-*m*W)⊗3Bz-3*n*W interactions, and underlying their enhancement and stability, we analyzed the interaction of a single benzene molecule hydrated with *n* water molecules in (M^+^-*m*W)⊗3Bz-3*n*W complexes. A hydrated benzene molecule participates in five kinds of interactions: (1) the interaction of the benzene molecule with the cation (denoted as *E*_M_^+^_-π_); (2) the interaction of the benzene molecule with other benzene molecules in the complex (denoted as *E*_π-π_); (3) the interaction of the water molecules with benzene molecules in the complex (denoted as *E*_W-π_); (4) the interaction of the water molecules around the cation or benzene molecule with other water molecules in the complex (denoted as *E*_W-W_); (5) the interaction of the water molecules with the cation (denoted as *E*_W-M_^+^). In this system, the benzene molecule of the hydrated benzene engages directly in *E*_M_^+^_-π_, *E*_π-π_, and *E*_W-π_ interactions and is indirectly influenced by *E*_W-W_ and *E*_W-M_^+^. The water molecules of the hydrated benzene, on the other hand, are directly involved in *E*_W-π_, *E*_W-W_, and *E*_W-M_^+^ interactions while being indirectly affected by *E*_M_^+^_-π_ and *E*_π-π_ interactions.

**Figure 4:**
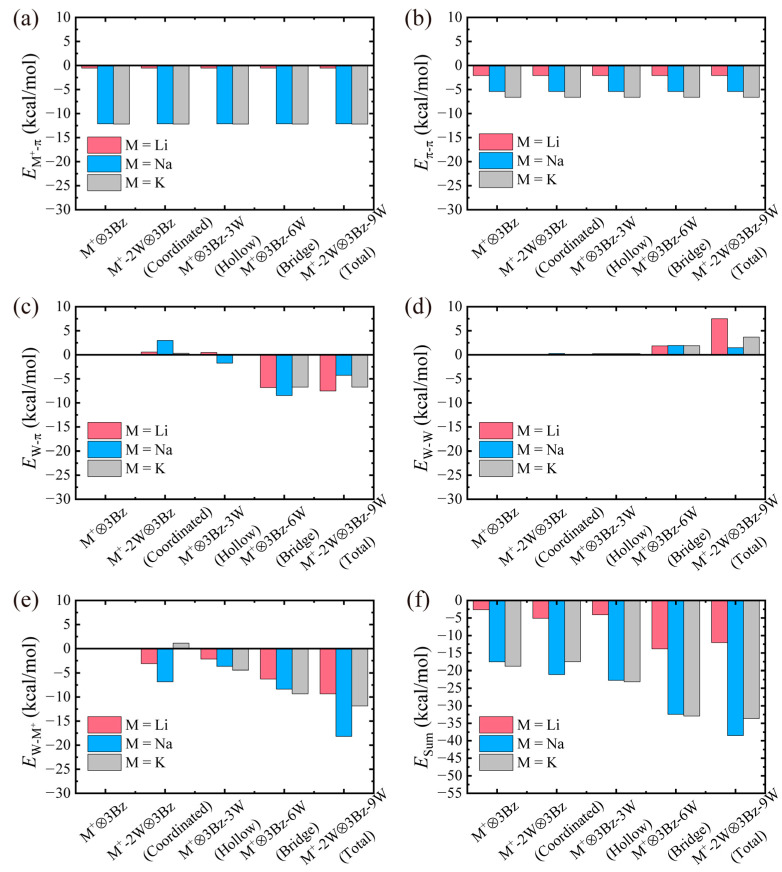
Separation of cation⊗3π interaction energy. The values are partitioned into (**a**) cation–π interaction energy (*E*_M_^+^**_-_**_π_), (**b**) π–π interaction energy (*E*_π-π_), (**c**) water–π interaction energy (*E*_W-π_), (**d**) water–water interaction energy (*E*_W-W_), (**e**) water–ion interaction energy (*E*_W-M_^+^), and (**f**) their summation (*E*_Sum_).

We defined cation–π interaction energy (*E*_M_^+^_-π_) as *E*_M_^+^_-π_ = *E*_M_^+^_-3π_ − *E*_M_^+^_-2π_. *E*_M_^+^_-3π_ = *E*(M^+^⊗3Bz) − *E*(M^+^) − *E*(3Bz) denotes the interaction between the cation and the three benzene molecules. Similarly, *E*_M_^+^_-2π_ = *E*(M^+^⊗2Bz) − *E*(M^+^) − *E*(2Bz) denotes the interaction between the cation and the two benzene molecules. *E*(3Bz) and *E*(2Bz) are the energy of three or two benzene molecules in (M^+^-*m*W)⊗3Bz-3*n*W or (M^+^-*m*W)⊗2Bz-2*n*W complexes in the absence of the cation and water molecules (if *n* ≠ 0). We thereby obtained the cation–π interaction energy of an individual benzene molecule with the cation while taking into account the effect from the other benzene molecules in the complex (see Table 2 and Figure 4a), which is consistent with the definition in previous studies [1]. Table 2 and Figure 4a show that the strength of *E*_M_^+^_-π_ (the absolute value) increases in the order of Li^+^ (−0.54 kcal/mol) < Na^+^ (−12.12 kcal/mol) < K^+^ (−12.17 kcal/mol) for all (M^+^-*m*W)⊗3Bz-3*n*W complexes. The *E*_Na_^+^_-π_ and *E*_K_^+^_-π_ are nearly degenerate, and their contributions to *E*_Sum_ are significantly larger than those of other energy components. In contrast, the *E*_Li_^+^_-π_ is weak and contributes minimally to *E*_Sum_. These results suggest that ions with larger ionic radii, such as Na^+^ and K^+^, make closer contact with the benzene molecules in the aromatic box, leading to stronger interactions. We note that the atomic positions of all benzene molecules and the cation were fixed to ensure that the structure formed by the benzene molecules remained consistent with their gas-phase configuration. As a result, the *E*_M_^+^_-π_ values for all complexes are identical.

Then, we calculated the π–π interaction energy of a single benzene with respect to the other benzenes in the complex as *E*_π-π_ = *E*_3π_ − *E*_2π_ with *E*_3π_ = *E*(3Bz) − 3*E*(Bz) and *E*_2π_ = *E*(2Bz) − 2*E*(Bz). Here, *E*(Bz) represents the energy of a benzene molecule. The results of the π–π interaction energy allowed us to evaluate the effect from the relative positions of the benzenes in (M^+^-*m*W)⊗3Bz-3*n*W complexes (see Table 2 and Figure 4b). The strength of *E*_π-π_ (the absolute value) increases in the order of Li^+^ (−2.08 kcal/mol) < Na^+^ (−5.41 kcal/mol) < K^+^ (−6.62 kcal/mol) for all (M^+^-*m*W)⊗3Bz-3*n*W complexes. This indicates that ions with larger ionic radii enable more favorable steric arrangements, resulting in stronger *E*_π-π_. Here, due to the atomic positions of all benzene molecules and the cation being fixed, the *E*_π-π_ values for all complexes are identical.

Next, we calculated the water–π interaction energy of the water molecules around the cation or benzene molecule with respect to the other benzene molecules in the complex as *E*_W-π_ = *E*_W-3π_ − *E*_W-2π_ with *E*_W-3π_ = *E*(*m*W⊗3Bz-3*n*W) − *E*(3Bz) − *E*(*m*W-3*n*W) and *E*_W-2π_ = *E*(*m*W⊗2Bz-2*n*W) − *E*(2Bz) − *E*(*m*W-2*n*W). *E*(*m*W⊗3Bz-3*n*W) and *E*(*m*W⊗2Bz-2*n*W) are the energies of the (M^+^-*m*W)⊗3Bz-3*n*W and (M^+^-*m*W)⊗2Bz-2*n*W complexes in the absence of the cation, respectively. *E*(*m*W-3*n*W) and *E*(*m*W-2*n*W) are the energies of the (M^+^-*m*W)⊗3Bz-3*n*W and (M^+^-*m*W)⊗2Bz-2*n*W complexes in the absence of the cation and benzene molecules, respectively. *E*_W-3π_ and *E*_W-2π_ denote the interaction between the water molecules and the (three or two) benzene molecules. *E*_W-π_ is the interaction energy of the water molecules around an individual benzene molecule with the other benzene molecules while taking into account the effect of the other water molecules in the complex (see Table 2 and Figure 4c). The change in the strength of *E*_W-π_ is quite small except when *n* = 2 and 3, suggesting that the water–π interaction becomes significant only when the water molecule is adsorbed at the bridge site. In addition, at *n* = 2 and 3, the contribution of *E*_W-π_ to *E*_Sum_ is relatively large, particularly in the case of Li^+^. At this point, the strength of *E*_W-π_ is independent of the ion species, suggesting that it is not influenced by the presence of the ions. This is expected, as the water molecule interacts exclusively with the benzene molecule.

The interaction energy (*E*_W-W_) of the water molecules around the cation or benzene molecule with respect to the other water molecules in the complex was defined as *E*_W-W_ = *E_m_*_W-3*n*W_ − *E_m_*_W-2*n*W_ with *E_m_*_W-3*n*W_ = *E*(*m*W-3*n*W) − (*m* + 3*n*)*E*(W) and *E_m_*_W-2*n*W_ = *E*(*m*W-2*n*W) − (*m* + 2*n*)*E*(W). Here, *E*(W) represents the energy of a water molecule. *E*(*m*W-3*n*W) and *E*(*m*W-2*n*W) are the energies of *m* + 3*n* or *m* + 2*n* water molecules in (M^+^-*m*W)⊗3Bz-3*n*W or (M^+^-*m*W)⊗2Bz-2*n*W complexes in the absence of the cation and benzene molecules. *E*_W-W_ allowed us to evaluate the effect from the relative positions of the water molecules in (M^+^-*m*W)⊗3Bz-3*n*W complexes (see Table 2 and Figure 4d). The change in the strength of *E*_W-W_ is quite small except when water molecules occupy bridge sites (*n* = 2 and 3). In addition, the values of *E*_W-W_ are positive, such as 7.49 kcal/mol for Li^+^-2W⊗3Bz-9W, indicating that the water–water interaction weakens the stability of the cation⊗3π system.

The interaction energy (*E*_W-M_^+^) of the water molecules around the cation or benzene molecule with respect to the cation in the complex was defined as *E*_W-M_^+^ = *E*_M_^+^_-*m*W-3*n*W_ − *E*_M_^+^_-*m*W-2*n*W_ with *E*_M_^+^_-*m*W-3*n*W_ = *E*(M^+^-*m*W-3*n*W) − *E*(*m*W-3*n*W) − *E*(M^+^) and *E*_M_^+^_-*m*W-2*n*W_ = *E*(M^+^-*m*W-2*n*W) − *E*(*m*W-2*n*W) − *E*(M^+^). Here, *E*(M^+^) represents the energy of a cation. *E*(M^+^-*m*W-3*n*W) and *E*(M^+^-*m*W-2*n*W) are the energies of the cation with *m* + 3*n* or *m* + 2*n* water molecules in (M^+^-*m*W)⊗3Bz-3*n*W or (M^+^-*m*W)⊗2Bz-2*n*W complexes in the absence of benzene molecules. We thereby obtained the W-M^+^ interaction energy of the water molecules bound to the cation or the individual benzene molecule with the cation while taking into account the effect from the other water molecules in the complexes (see Table 2 and Figure 4e). As water molecules directly bind to the cation (coordinated site), *E*_W-Li_^+^ (−3.11 kcal/mol) in the Li^+^-2W⊗3Bz complex and *E*_W-Na_^+^ (−6.82 kcal/mol) in Na^+^-2W⊗3Bz are a negative sign, while *E*_W-K_^+^ (1.13 kcal/mol) in K^+^-2W⊗3Bz is a positive sign. It suggests that the contribution of hydrated Li^+^ and hydrated Na^+^ to the structural stability of the M^+^-2W⊗3Bz complex is greater than that of hydrated K^+^. Furthermore, as water molecules hydrate benzene molecules (hollow and bridge sites), the values of *E*_W-M_^+^ are negative, showing positive contributions to the structural stability of M^+^⊗3Bz-3W and M^+^⊗3Bz-6W complexes, and their strengths increase in the order of Li^+^ < Na^+^ < K^+^. Moreover, the contribution of *E*_W-M_^+^ to *E*_Sum_ is relatively significant, particularly in the case of M^+^-2W⊗3Bz-9W.

Now, we can rewrite Equation (1) as

(2) $$E_{\mathrm{Sum}}\left(\left(M^{+}-mW\right)⊗3\mathrm{Bz}-3nW\right)=E_{M^{+}-\pi }+E_{\pi -\pi }+E_{W-\pi }+E_{W-W}+E_{{W-M}^{+}}$$

As shown in Table 2, when comparing *E*_Sum_ and *E*_binding_, we find that they are relatively close only when M = K. However, for M = Li or Na, the difference between *E*_Sum_ and *E*_binding_ in M^+^-2W⊗3Bz is significant, with *E*_Sum_ being significantly overestimated (more negative). Similarly, the discrepancy between *E*_Sum_ and *E*_binding_ is also pronounced for Na^+^-2W⊗3Bz-9W, with *E*_Sum_ again being substantially overestimated. These large discrepancies suggest that the separation of the cation⊗3π interaction energy should only be considered a rough estimation method. Therefore, we can only roughly discuss the origin of the stabilization of (M^+^-*m*W)⊗3Bz-3*n*W complexes by the mechanism analyses. Five types of energy values are shown in Figure 4 to easily look at what factors determine the stability of (M^+^-*m*W)⊗3Bz-3*n*W complexes. It is clear that the cation–π interaction energies (*E*_M_^+^**_-_**_π_) are always a key in determining the stability of (M^+^-*m*W)⊗3Bz-3*n*W complexes except for M = Li, ranging from −12.12 to −12.17 kcal/mol for M = Na and K, respectively (Figure 4 and Table 2), are relatively significant. We note that the larger the value of the interaction energy, the weaker its strength. A more striking finding in Figure 4a is that the strength of cation–π interaction for M = Li is quite smaller (−0.54 kcal/mol), even less than π–π, water–π, and water–ion interactions at *n* = 2 and 3. Overall, Table 2 shows that in the presence of surrounding water molecules, the stability of (M^+^-*m*W)⊗3Bz-3*n*W complexes is generally enhanced by cation–π, π–π, water–π, and water–ion interactions, while water–water interactions typically have a destabilizing effect.

### 2.3. SAPT Analysis

Considering that the OBB energy of the cation⊗3π interaction corresponds to the binding energy of a single benzene molecule, we divided the (M^+^-*m*W)⊗3Bz-3*n*W complex (*m* = 0 or 2, and *n* = 0, 1, or 2) into two parts: (1) (M^+^-*m*W)⊗2Bz-2*n*W and (2) Bz-*n*W. We then employed the symmetry-adapted perturbation theory (SAPT) to calculate the decomposition (*E*_elst_, *E*_exch_, *E*_ind_, *E*_disp_) of the interaction energy (*E*_tot_) between these two parts using geometries optimized at the MP2/6-311++G(d,p) level, as shown in Figure 2. Here, *E*_tot_ = *E*_elst_ + *E*_exch_ + *E*_ind_ + *E*_disp_, where *E*_elst_, *E*_exch_, *E*_ind_, and *E*_disp_ are polarization terms, namely electrostatics, exchange, induction, and dispersion, respectively. *E*_elst_ arises from Coulombic multipole–multipole interactions and the overlap between monomeric charge distributions. *E*_exch_ is a consequence of the spatial overlap of the monomer wavefunctions and the anti-symmetry requirement of the dimer wavefunction following the exchange of electronic coordinates. *E*_ind_ is due to the polarization response of the monomers to each other’s electric fields, as well as the charge transfer between them. *E*_elst_ originates from the correlation between the electrons of the monomers.

Firstly, it is important to note that when calculating the OBB energy of the cation⊗3π interaction, the subtracted energy originates from the optimized (M^+^-*m*W)⊗2Bz-2*n*W complex. In contrast, when calculating *E*_tot_, the structure of the (M^+^-*m*W)⊗2Bz-2*n*W complex is directly taken from the (M^+^-*m*W)⊗3Bz-3*n*W complex. The energy of the optimized structure at the same computational level should be significantly lower than that of the non-optimized structure. Consequently, the calculated OBB energy (Table 1) is significantly larger than *E*_tot_ (Table 3). Even though there is a considerable discrepancy between the binding energies obtained by the two methods, SAPT analysis not only provides insight into the interaction energies between the two parts but also allows us to determine which physically meaningful components predominantly contribute to the interactions.

Table 3 shows that compared to the M^+^⊗3Bz complex, the *E*_tot_ of M^+^-2W⊗3Bz (coordinated) significantly increases when water molecules are directly coordinated to the cation. Conversely, the *E*_tot_ of M^+^⊗3Bz-3W (hollow) and M^+^⊗3Bz-6W (bridge) significantly decreases when water molecules are directly adsorbed onto the benzene molecules. This change is similar to the corresponding change in OBB energy (Table 1). Specifically, the increase in *E*_tot_ for M^+^-2W⊗3Bz (coordinated) is mainly due to contributions from *E*_elst_, *E*_exch_, and *E*_ind_, while *E*_disp_ decreases. This indicates that the direct coordination of water molecules to the cation enhances the correlation between the electrons of the two parts but weakens the Coulombic multipole–multipole interactions, charge transfer, and electric exchange between the two parts. Again, we note that the lower the value of the interaction energy, the greater its strength.

Furthermore, Table 3 shows that the decrease in *E*_tot_ for M^+^⊗3Bz-3W (hollow) and M^+^⊗3Bz-6W (bridge) is primarily due to contributions from *E*_elst_ and *E*_disp_, with *E*_elst_ contributing the most, while *E*_exch_ and *E*_ind_ increase. This suggests that the direct adsorption of water molecules onto the benzene molecules significantly enhances the Coulombic multipole–multipole interactions between the two parts and slightly enhances the correlation between the electrons of the two parts. Additionally, the direct adsorption of water molecules onto the benzene molecules weakens the charge transfer and electric exchange between the two parts. Interestingly, in both cases—whether water molecules are directly coordinated to the cation or adsorbed onto the benzene molecules—*E*_exch_, *E*_ind_, and *E*_disp_ exhibit the same directional changes. However, the value of *E*_elst_ increases (indicating a weaker interaction) when water molecules are coordinated to the cation and decreases (indicating a stronger interaction) when they are adsorbed onto benzene. These findings suggest that the primary effect of water molecules at different adsorption sites is to modulate the Coulombic multipole–multipole interactions and the overlap between monomeric charge distributions, thereby influencing the changes in OBB energy.

### 2.4. Molecular Dynamic Simulations

Now, we employed molecular dynamics (MD) simulations to highlight the significance of cation⊗3π interactions. Alkali metal ions, particularly Na^+^ ions, are ubiquitous in biological systems. When Na^+^ interacts with the three-aromatic-ring motif of a protein, we expect that the strong cation⊗3π interactions will enhance the structural stability of the motif, thereby influencing its associated biological functions. To test this hypothesis, we performed ab initio molecular dynamics (AIMD) simulations on a small motif comprising a few dozen atoms. This approach was necessary because classical MD simulations, which can handle protein systems with tens of thousands of atoms, currently lack force fields capable of accurately describing the interactions between tryptophan residues and Na^+^ ions [21]. Although force fields for phenylalanine–Na^+^ interactions have been proposed [22], the absence of comprehensive force fields led us to reduce the model size for first-principles simulations.

**Figure 5:**
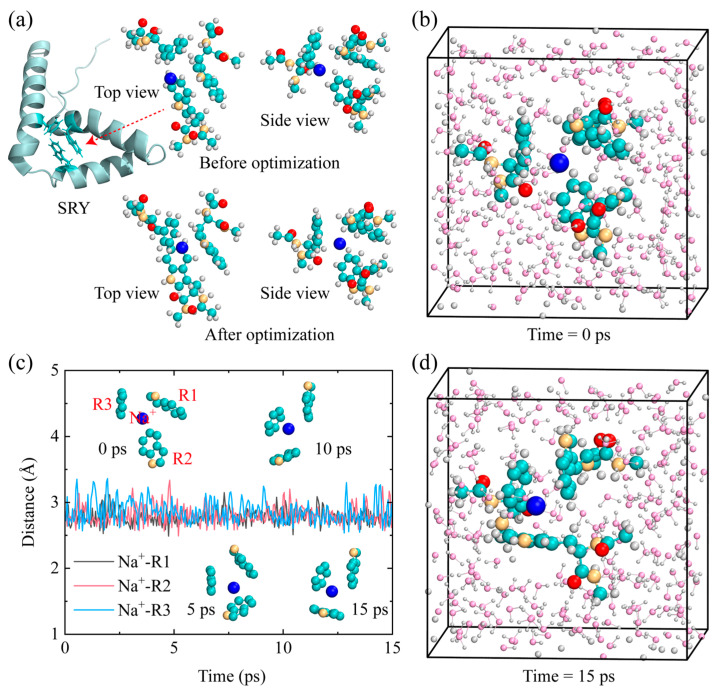
(**a**) Structure of the SRY protein and its three-aromatic-ring motif (shown as dark cyan), together with a Na^+^ ion positioned near the motif, shown before and after structural optimization. (**b**) Initial configuration of the AIMD simulations at 0 ps. (**c**) Distances between Na^+^ and rings as a function of simulation time from the AIMD simulations, where the distance is defined as the shortest distance between the cation and any carbon or nitrogen atom in the aromatic rings. (**d**) Snapshot from the AIMD simulations at 15 ps. Spheres in blue, cyan, orange, red, white, and pink represent Na^+^, carbon, nitrogen, oxygen in motif, hydrogen, and oxygen in water, respectively.

We selected the three-aromatic-ring motif from the sex-determining region Y (SRY) protein, consisting of two tryptophan residues and one phenylalanine residue. The three-aromatic-ring motif within this protein plays a key role in its biological functions [23], making it an ideal model to explore how cation⊗3π interactions stabilize protein structures and influence their activity. A Na^+^ ion was then placed randomly near the aromatic rings, and the structure was optimized, as shown in Figure 5a. To realistically reflect the motion of large protein molecules, which is much slower than that of ions or water molecules, we fixed the carbon atom of the methyl group located at the opposite end of the aromatic rings. All atoms in the aromatic rings and their connecting side chains were allowed to move freely. As expected, after structural optimization, the Na^+^ ion became surrounded by the three aromatic rings (Figure 5a).

Next, the optimized configuration, consisting of the three-aromatic-ring motif and the Na^+^ ion, was placed in a 20 × 20 × 20 Å^3^ box containing 194 water molecules with a density of 1.0 g/cm^3^ for AIMD simulations at 298 K (details provided in the Methods section). The initial configuration is shown in Figure 5b. During the simulation, the Na^+^ ion remained within a distance of 2.5–3.5 Å from the three aromatic rings (Figure 5c), as illustrated by the inset structure showing a stable triangular arrangement, ◬. At 15 ps, the Na^+^ ion is fully surrounded by the three aromatic rings, forming a stable triangular structure (Figure 5d). These results indicate that, even under thermal perturbations at 298 K, the cation⊗3π configuration between the three-aromatic-ring motif of the SRY protein and the Na^+^ ion remains stable. This underscores the biological significance of our findings, highlighting the potentially crucial role of cation⊗3π interactions in stabilizing protein structures.

## 3. Methods

First-principles calculations: All first-principles calculations were carried out using the second-order Møller–Plesset perturbation theory (MP2) with the 6-311++G(d, p) basis set of triple-zeta quality and including diffuse functions applied on all atoms. We note that MP2 has been widely used to study cation–π interaction between aromatic ring structures and cations [24,25,26,27]. In order to minimize disruption to the symmetric benzene scaffold and accurately investigate the role of water molecule adsorption sites in modulating the strength of cation⊗3π interactions, we employed a unique structural optimization strategy. Initially, for complexes without water molecules, all atoms were allowed to move freely during structural optimization, with no symmetry constraints imposed. Subsequently, when adding water molecules at three different hydration sites (denoted as coordinated, hollow, and bridge), we fixed the atomic positions of all benzene molecules and the cation to ensure that the structure formed by benzene molecules remains consistent with its gas-phase configuration. In addition, we also constrained the angle between the water molecule, benzene molecule, and cation during structural optimization to ensure the water molecules remained at the desired hydration sites. For the configurations formed by the three hydration sites (denoted as Total), we performed single-point energy calculations only. The total charge of each system containing alkali cations was +1e. Geometries of all configurations were optimized at the MP2/6-311++G(d, p) level of theory with tight SCF (10^−7^ *E*_h_) and default geometry convergence. All first-principles calculations based on MP2 were performed using the ORCA quantum chemistry package (version 4.2.1) [28,29]. All symmetry adapted perturbation theory (SAPT) calculations were carried out with the PSI4 program (version 1.9.1) [30] with the DF technique.

AIMD simulations: The ab initio molecular dynamics (AIMD) simulations were conducted in an NVT ensemble at a constant temperature of 298 K, controlled by a Nosé thermostat. A time step of 1.0 fs was employed. The electronic parameters for both the simulations and geometry optimizations were set consistently, with a self-consistent charge (SCC) tolerance of 1 × 10^−5^ kcal/mol. No symmetry constraints were applied during either the simulations or the geometry optimizations. All energy and force calculations were performed using the DFTB+ method, incorporating SCC and dispersion corrections, along with the 3ob-3-1 Slater-Koster parameter set [31,32]. As a semiempirical version of density functional theory (DFT), DFTB+ offers reliable computational efficiency, providing accurate results for the structure and stability of large molecular systems with significantly reduced computational cost compared to standard DFT [33,34]. All AIMD simulations and geometry optimizations for the three-aromatic-ring motif derived from the SRY protein were carried out using the DFTB+ program (version 17.1) [35].

## 4. Conclusions

In summary, first-principles calculations were carried out to study the effect of hydration on cation⊗3π interactions. We found that the cation⊗3π interaction energy is sensitive to the site of solvation of cation⊗3π systems. Solvation of the metal cation decreases the strengths of cation⊗3π interactions, while the solvation of the benzene molecule increases the strengths of cation⊗3π interactions, compared with the strengths of cation⊗3π interactions in the gas phase. The enhancement of the cation⊗3π interactions by solvation agrees with the observation in previous studies that the solvation of benzene increases its interaction energy with the metal ion (cation–π interactions) [17]. The mechanism analyses revealed that in the presence of surrounding water molecules, the stability of cation⊗3π systems is generally enhanced by cation–π, π–π, water–π, and water–ion interactions, while water–water interactions typically have a destabilizing effect.

The underlying physics in the behaviors of the cation⊗3π interactions was further explored by SAPT analysis, and we find that the primary effect of water molecules at different adsorption sites is to modulate the Coulombic multipole–multipole interactions and the overlap between monomeric charge distributions, thereby influencing the changes in OBB energy of cation⊗3π interaction. Moreover, the strengths of cation⊗3π interactions in the presence of solvents follow the order Li^+^ < Na^+^ < K^+^. The ion size dependence of cation⊗3π interactions suggests potential application for morphologic selectivity self-assembly of bio/nanomaterials in aqueous solutions containing different metal cations. In the case of biological molecules, it was found that the behavior of protein, including its aggregate behavior, in pure water can be very different from its behavior in phosphate buffer [36]. Moreover, aromatic rings are rich in biological systems and carbon-based materials. Our AIMD simulations underscore the practical significance of cation⊗3π interactions, particularly within structural arrangements such as the three-aromatic-ring motif, which is essential for the functionality of proteins like the SRY protein. Previous experimental studies demonstrate that the three-aromatic-ring motif in the SRY protein is crucial for specific DNA binding and bending [23]. Therefore, the enhancement of the cation⊗3π interactions in the presence of surrounding solvent emphasizes their important role in the expression of the biological function in biological systems and the design of carbon-based materials.

## Figures and Tables

**Table 1:** OBB energies (*E*_binding_) of cation⊗3π interaction obtained at the MP2/6-311++G(d, p) level (all values in kcal/mol).

M	M^+^⊗3Bz		M^+^-2W⊗3Bz (Coordinated)	M^+^⊗3Bz-3W (Hollow)	M^+^⊗3Bz-6W (Bridge)	M^+^-2W⊗3Bz-9W (Total)
Li	−2.63	1.31 ^(a)^	−1.31	−4.93	−12.06	−8.31
Na	−17.53	−12.08 ^(a)^	−11.18	−20.84	−28.21	−26.44
K	−18.78	−14.60 ^(a)^	−15.24	−23.03	−30.93	−29.06

^(a)^ OBB energy taken from Ref. [1].

**Table 2:** M^+^–π interaction, π–π interaction, W–π interaction, W-W interaction, and W-M^+^ interaction energies in (M^+^-*m*W)⊗3Bz-3*n*W complexes (all values in kcal/mol).

Complexes	*E*_M_^+^_-π_	*E*_π-π_	*E*_W-π_	*E*_W-W_	*E*_W-__M_^+^	*E*_Sum_	*E*_binding_^a^
Li^+^⊗3Bz	−0.54	−2.08	0.00	0.00	0.00	−2.63	−2.63
Li^+^-2W⊗3Bz	−0.54	−2.08	0.57	0.06	−3.11	−5.10	−1.31
Li^+^⊗3Bz-3W	−0.54	−2.08	0.48	0.25	−2.17	−4.07	−4.93
Li^+^⊗3Bz-6W	−0.54	−2.08	−6.80	1.84	−6.26	−13.84	−12.06
Li^+^-2W⊗3Bz-9W	−0.54	−2.08	−7.53	7.49	−9.33	−12.00	−8.31
Na^+^⊗3Bz	−12.12	−5.41	0.00	0.00	0.00	−17.53	−17.53
Na^+^-2W⊗3Bz	−12.12	−5.41	2.97	0.26	−6.82	−21.11	−11.18
Na^+^⊗3Bz-3W	−12.12	−5.41	−1.76	0.25	−3.68	−22.72	−20.84
Na^+^⊗3Bz-6W	−12.12	−5.41	−8.46	1.93	−8.37	−32.43	−28.21
Na^+^-2W⊗3Bz-9W	−12.12	−5.41	−4.26	1.48	−18.18	−38.50	−26.44
K^+^⊗3Bz	−12.17	−6.62	0.00	0.00	0.00	−18.78	−18.78
K^+^-2W⊗3Bz	−12.17	−6.62	0.32	−0.13	1.13	−17.47	−15.24
K^+^⊗3Bz-3W	−12.17	−6.62	−0.15	0.25	−4.47	−23.16	−23.03
K^+^⊗3Bz-6W	−12.17	−6.62	−6.71	1.88	−9.34	−32.95	−30.93
K^+^-2W⊗3Bz-9W	−12.17	−6.62	−6.73	3.67	−11.84	−33.68	−29.06

^a^ OBB energy is defined in Equation (1).

**Table 3:** Contributions to the interaction energy (*E*_tot_) between the (M^+^-*m*W)⊗2Bz-2*n*W and Bz-*n*W complexes obtained by SAPT calculations (all values in kcal/mol).

Complexes	M	*E*_elst_	*E*_exch_	*E*_ind_	*E*_disp_	*E*_tot_
M^+^⊗3Bz	Li	−70.49	109.10	−39.22	−59.63	−60.24
	Na	−58.62	72.76	−30.32	−44.88	−61.07
	K	−47.40	46.21	−24.95	−35.82	−61.96
M^+^-2W⊗3Bz (Coordinated)	Li	−65.03	112.67	−32.56	−65.26	−50.18
	Na	−56.10	102.77	−21.78	−58.78	−33.88
	K	−40.76	61.45	−17.17	−45.40	−41.89
M^+^⊗3Bz-3W (Hollow)	Li	−88.27	114.20	−37.59	−61.48	−73.14
	Na	−75.86	76.34	−28.94	−46.38	−74.84
	K	−63.36	48.39	−24.07	−37.05	−76.09
M^+^⊗3Bz-6W (Bridge)	Li	−94.87	111.56	−36.37	−63.36	−83.03
	Na	−82.90	74.62	−27.93	−47.94	−84.15
	K	−70.52	47.22	−23.06	−38.09	−84.45

## Data Availability

Data will be made available upon request.
